# Moderate‐Intensity Intermittent Walking Improves Liver‐Related Biomarkers and Reduces Inflammation in Postmenopausal Women With Obesity: A Randomized Controlled Study

**DOI:** 10.1002/ejsc.70147

**Published:** 2026-02-24

**Authors:** Wissal Abassi, Nejmeddine Ouerghi, Antonella Muscella, Moncef Feki, Anissa Bouassida

**Affiliations:** ^1^ Research Unit “Sport Sciences, Health and Movement”(UR22JS01) High Institute of Sport and Physical Education of Kef University of Jendouba Kef Tunisia; ^2^ Faculty of Medicine of Tunis University of Tunis el Manar Rabta Hospital Tunis Tunisia; ^3^ University of Gafsa High Institute of Sport and Physical Education of Gafsa Gafsa Tunisia; ^4^ Department of Biological and Environmental Science and Technologies (DiSTeBA) University of Salento Lecce Italy

**Keywords:** 6‐minute walk test, alanine transaminase, alkaline phosphatase, C‐reactive protein, erythrocyte sedimentation rate, postmenopausal obesity, walking training

## Abstract

Postmenopausal obesity is an important public health concern, accompanied by increased systemic inflammation that heightens the risk of liver disease. Exercise improved both inflammatory and hepatic function. Moderate‐intensity intermittent‐walking training (MIWT) is considered a feasible approach for postmenopausal women with obesity. This study aimed to investigate whether MIWT could reduce the risk by modulating hepatic enzymes and selected inflammatory markers. Thirty‐six sedentary postmenopausal women with obesity (mean age 55.7 ± 3.5 years; mean weight: 86.9 ± 12.2 kg; mean BMI: 34.0 ± 5.0 kg/m^2^) were randomly assigned to a training group (TG, *n* = 18) or a control group (CG, *n* = 18). The TG completed a 10‐week MIWT protocol (4 sessions/week, ∼85 min/session), involving repeated walking intervals at 60%–80% of the 6MWT distance with active recovery. As a secondary objective, changes in body composition and aerobic capacity were also assessed. Significant improvements were observed in the TG group in liver enzymes alanine‐transaminase (ALT), (*p* = 0.002, *d* = 0.29), aspartate‐transaminase (AST) (*p* = 0.013, *d* = 0.29), gamma‐glutamyl‐transferase (GGT) (*p* = 0.036, *d* = 0.25), total bilirubin (*p* = 0.009, *d* = 0.13), and C‐reactive‐protein (CRP) (*p* = 0.007, *d* = 0.49). Additionally, significant reductions were found in body mass (*p* < 0.001), BMI (*p* < 0.001), body fat (*p* = 0.001), and waist circumference (*p* < 0.001), along with increased aerobic capacity (*p* = 0.031). These findings indicate that MIWT is a feasible and effective intervention for inducing favorable changes in liver‐related biochemical markers and systemic inflammation, with additional benefits for body composition and aerobic fitness in postmenopausal women with obesity.

## Summary

- Moderate‐intensity intermittent walking (MIWT) significantly improved liver enzymes (ALT, AST, GGT) and increased total bilirubin, indicating enhanced hepatic function in postmenopausal women with obesity.
- Systemic inflammation was reduced, as evidenced by significant decreases in C‐reactive protein (CRP) following the 10‐week MIWT program.
- MIWT also improved body composition by reducing body mass, BMI, body fat, and waist circumference, while enhancing aerobic capacity.
- MIWT represents a feasible, low‐cost, non‐pharmacological intervention that can be integrated into community‐ or primary care–based programs to improve metabolic health in postmenopausal women with obesity.

## 1 Introduction

The postmenopausal stage of life is a physiological event accompanied by hormonal changes, that are associated with an array of metabolic changes (Khalafi et al. 2021), including weight gain, insulin resistance, lipid abnormalities, and proinflammatory response (Goossens et al. 2021). Declines in estrogen levels increased systemic inflammation, marked by the increased expression of pro‐inflammatory cytokines—interleukin‐6, tumor necrosis factor alpha, C‐reactive protein (CRP), as well as an increase in erythrocyte sedimentation rate (ESR) (Suyasa et al. 2018; Au et al. 2016). These events generally increase the risk of cardiometabolic and liver diseases (Goossens et al. 2021). At high levels, CRP is associated with reduced liver function in individuals with obesity (Zimmermann et al. 2011). Additionally, higher CRP is linked to elevations of liver enzymes leading to the development of liver dammage (Kuroda et al. 2021). Moreover, the ESR has been shown to be increased in patients with liver diseases when compared to healthy participants (Das et al. 2011).

Although conditions such as nonalcoholic fatty liver disease (NAFLD) are more prevalent among postmenopausal women and individuals with obesity and are often accompanied by elevations in hepatic enzymes (Younossi et al. 2016; Teng et al. 2023; Estes et al. 2018; Pacifico et al. 2014), our use of these references serves solely to contextualize the clinical relevance of monitoring liver‐related biomarkers in populations with increased metabolic risk. Current pharmacological and hormonal therapies show inconsistent effectiveness and may be associated with adverse effects, underscoring the growing need for safe, accessible, and non‐pharmacological strategies to support hepatic and metabolic health in this population (Borrelli and Ernst 2010; DiStefano 2020).

Therefore, there is a growing interest in potential alternative strategies among postmenopausal women. Lifestyle modification based on regular physical activity is the most attractive option for many obese (Abassi et al. 2020, 2022, 2023, 2025) and postmenopausal women (Son et al. 2023). Aerobic training improves body composition and inflammatory markers in postmenopausal women with obesity (Son et al. 2023). A systematic review and meta‐analysis showing that aerobic training lasting longer than 8 weeks is an effective intervention for reducing CRP in postmenopausal women struggling with overweight and obesity (Tan et al. 2023). Literature is, however, scarce regarding the possible benefits of aerobic training in liver enzymes in postmenopausal women. One study revealed that liver enzymes, including ALT, AST, ALP, and GGT, did not change significantly after 24 weeks of aerobic physical activity in postmenopausal women with obesity (Rezende et al. 2016). Among various aerobic exercises, walking is the most recommended for post‐menopausal women with obesity to manage overall health. Moreover, it was demonstrated that postmenopausal women with obesity preferred moderate intermittent walking because it did not feel boring or painful (Coquart et al. 2012), which has been reported to be attractive in preventing cardiovascular risk in this population (Son et al. 2023).

The primary aim of this study was to investigate the effects of a 10‐week MIWT program on liver enzymes and selected inflammatory markers in postmenopausal women with obesity, given their clinical relevance for metabolic health. Secondary outcomes included changes in body composition and aerobic capacity, which are important indicators of overall physical function and health in this population. We hypothesized that participation in MIWT would result in significant improvements in liver‐related biomarkers and inflammatory profiles, accompanied by beneficial effects on body composition and aerobic fitness.

## 2 Methods

### 2.1 Study Design

This randomized controlled trial was conducted from September to December 2024. At the High Institute of Sport and Physical Education xxxxx. After baseline assessments of body composition, inflammatory markers, liver biomarker, and aerobic capacity, participants were randomly assigned to either the training group (TG) or the control group (CG) using a computer‐generated simple randomization sequence created with Microsoft Excel. No restrictions such as stratification or blocking were applied. Allocation was performed by an independent researcher not involved in participant assessment or data analysis to ensure allocation concealment and minimize selection bias. The randomization aimed to achieve balanced group allocation and comparable baseline characteristics between groups. To confirm group comparability at baseline, independent *t*‐tests and Mann‐Whitney U tests were performed, showing no statistically significant differences between groups for any of the baseline variables.

Following randomization, participants in the TG underwent a 10‐week moderate‐intensity intermittent walking training (MIWT) program, consisting of four sessions per week. Participants in the CG were instructed to maintain their regular daily routines and refrain from participating in any structured physical activity during the study period. No dietary restrictions were applied; however, all participants were asked to maintain their usual eating habits throughout the intervention period.

Attendance at all supervised training sessions was recorded, and participants received weekly reminders to enhance adherence. Outside the intervention, participants were instructed to maintain their usual physical activity and diet. Physical activity levels were self‐reported weekly using a questionnaire, while dietary intake was assessed by 3‐day food diaries collected at multiple time points and analyzed for energy intake and macronutrient composition.

All outcome measures were reassessed at week 11, upon completion of the intervention.

Adverse events were defined as any unintended physical effects or injuries related to the intervention. These were monitored systematically throughout the intervention period through direct observation by the supervising instructors and verbal questioning of participants at each session. No adverse events were reported during the study.

### 2.2 Ethics Declaration

The study was conducted in accordance with the Declaration of Helsinki and approved by the Scientific and Ethics Committee of XXX. All participants signed written informed consent after being informed of this investigation's aims, benefits, and risks. This trial was registered on ClinicalTrials.gov (https://clinicaltrials.gov/study/NCT06809270), on 05/02/2025.

### 2.3 Sample Size

**FIGURE 1:**
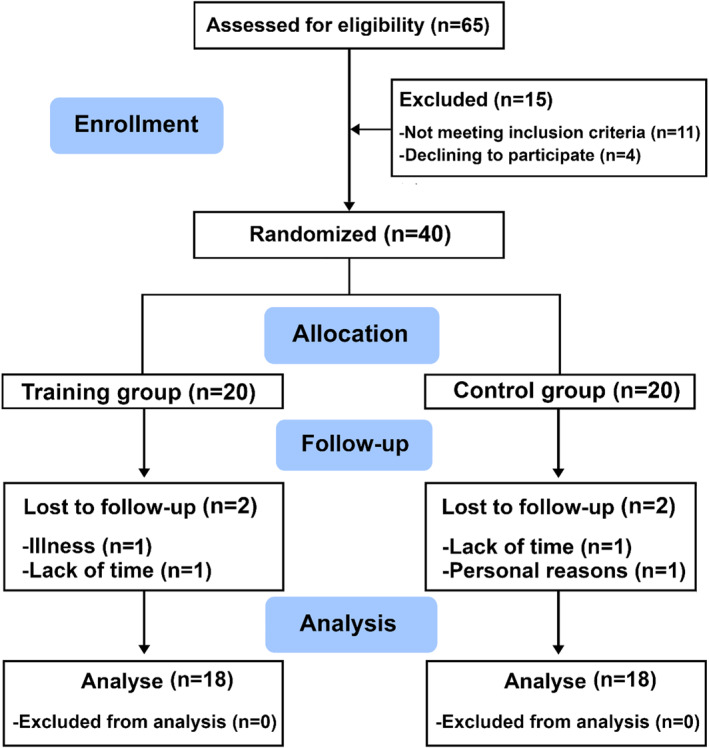
Flow‐chart of study.

We used G*Power 3.1 software to calculate the required sample size for a repeated‐measures ANOVA (2 groups × 2 time points), with a partial eta squared effect size of 0.55, alpha level of 0.05, and statistical power of 0.90. The analysis indicated that a minimum of 30 participants was needed. To account for potential dropouts, we enrolled 40 women. Four participants (2 from TG, 2 from CG) dropped out due to time constraints or personal reasons, resulting in a final sample of 36 participants (*n* = 18 per group) included in the per‐protocol analysis (see Figure 1).

### 2.4 Participants

A total of 65 women were screened for eligibility. Forty women meeting the inclusion criteria were enrolled and randomized into two groups (TG and CG). Of these, 36 participants (TG, *n* = 18; CG, *n* = 18) completed the study and were included in the final analysis (Figure 1). Four participants withdrew during the intervention (two from the TG and two from the CG) due to personal reasons unrelated to the program. No missing data were observed. The mean weight and BMI of participants were 86.9 ± 12.2 kg and 34.0 ± 5.0 kg/m^2^, respectively.

Adherence to the exercise program was high, with participants in the training group attending an average of 92% of the 40 prescribed sessions. Exercise intensity was individually prescribed based on the 6MWT distance and progressively increased from 60% to 80% of the baseline 6MWT over the 10‐week period. Compliance and intensity were monitored at each session using heart rate (Polar Electro Oy, Kempele, Finland) and the Borg Rating of Perceived Exertion (RPE) scale, ensuring participants exercised within the target moderate‐intensity range (60%–75% HRmax; RPE 12–14). No injuries or adverse events occurred during the study period. Overall, the exercise program was safe, well‐tolerated, and effectively implemented according to the prescribed protocol.

For the inclusion criteria, women had to be postmenopausal (≥ 1 year of amenorrhea, mean 4.5 ± 2.1 years, range 1–9 years), obese (BMI range 30–47 kg/m^2^), sedentary (< 120 min/week of low to moderate intensity physical activity during the past 6 months), and aged 50–60 years. Exclusion criteria included any cardiovascular, renal, pulmonary, or metabolic disease; systolic blood pressure ≥ 140 mmHg or diastolic blood pressure ≥ 90 mmHg; current use of medications or supplements affecting metabolic or cardiovascular function; menopausal hormone therapy; or orthopedic/musculoskeletal limitations interfering with exercise participation.

Before commencing the study, the participants were informed about potential risks and discomforts associated with the study.

Participants were instructed to maintain their usual diet throughout the 10‐week intervention. No specific dietary restrictions or interventions were applied. Nutritional status was monitored by asking participants to report any significant changes in their eating habits during the study period.

### 2.5 Body Composition and Aerobic Capacity Measurement

Height, body mass, and body fat percentage (BF) were determined with barefoot and lightly dressed subjects using a stadiometer (Holtain Ltd., Crymych, UK) and an electronic scale (Tanita BC‐533, Tokyo, Japan). The validity and reliability of this model for evaluating body composition in adults have been previously documented (Talma et al. 2013).

BMI (kg/m^2^) was calculated as body mass (kg) divided by height squared (m^2^). Waist (WC) and hip circumference (HC) were obtained at the midpoint between the superior border of the iliac crest and the lower border of the last rib and the maximum protuberance of the buttocks, respectively, using a measuring tape, at the end of a normal expiration. The waist–to–hip ratio (WHR) is the WC divided by HC. The 6‐min walk test was performed before and after the training intervention as an indicator of exercise capacity, according to the recommendations of the American Thoracic Society (ATS statement, 2002), in which women were asked to walk as fast as possible for 6 min on a 30‐m straight line and were allowed to stop or rest if necessary. The 6‐min walk test distance (6MWTdistance) was recorded at baseline and after the 10‐week intervention.

### 2.6 Exercise Intervention

**FIGURE 2:**
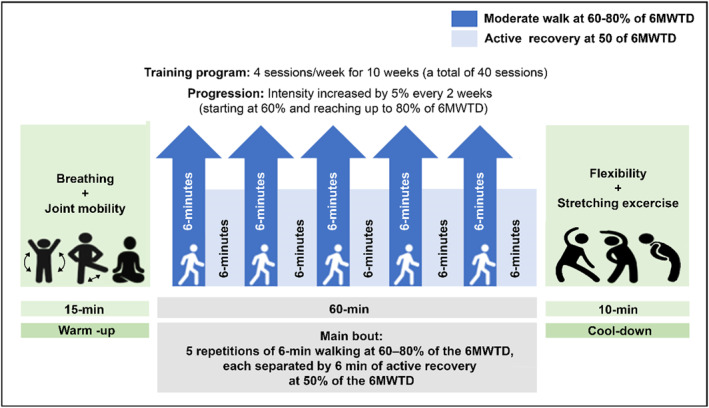
Schematic representation of the 10‐week moderate‐intensity intermittent walking training (MIWT) program.

A certified exercise specialist delivered the exercise protocol. The intervention consisted of a moderate‐intensity exercise program involving 4 sessions of walking per week, with a 24‐h washout period, for a total of 40 sessions, spanning 10 weeks. Each session carried out during this period includes a total of 3 well‐differentiated stages: a 15‐min standardized warm‐up in which slow walking combined with breathing exercises and joint mobility exercises were performed to avoid muscle and bone injuries; a 60‐min intermittent moderate‐intensity walking, consisting of a total of five repetitions of walking during 6 min at 60%–80% of the 6MWTdistance, with 6‐min active recovery between repetitions at 50% of the 6MWTdistance; a 10‐min cooling‐down part, in which flexibility exercises and stretching were performed. The exercise protocol began at 60% of participants at 60%–80% of the 6MWT distance and gradually increased by 5% every two weeks to reach 80% by week 10 (Figure 2). The training program was carried out according to Guessogo et al. (Guessogo et al. 2016) guidelines.

For set walking repetitions at 60%–80% of the 6MWT distance, each participant's baseline 6MWT distance was used to calculate target distances. For example, a 400 m 6MWT corresponds to 240 m (60%) in weeks 1–2. Participants completed five 6‐min walking repetitions per session, with 6‐min active recovery at 50% of the 6MWT distance. Intensity increased by 5% every 2 weeks. See Table 1 for the progression details. No concomitant care or co‐interventions were provided to participants during the trial.

**TABLE 1:** Example progression of walking distances over 10 weeks based on an illustrative 6MWT baseline distance of 400 m.

Weeks	% Of 6MWT distance	Target distance (Example: 6MWT = 400 m)	Active recovery distance (50% of 6MWT)	Estimated %HRmax range	Estimated %HRR range
1–2	60%	240 m	200 m	60%–65%	50%–55%
3–4	65%	260 m	200 m	63%–68%	52%–58%
5–6	70%	280 m	200 m	65%–70%	55%–60%
7–8	75%	300 m	200 m	68%–72%	58%–63%
9–10	80%	320 m	200 m	79%‐75%	60%–65%

*Note:* The table provides an example based on a 6MWT baseline distance of 400 m. Actual walking distances and intensity ranges were individually adjusted according to each participant's baseline 6MWT result. Exercise intensity was prescribed and monitored to maintain moderate effort (60%–75% HRmax, corresponding approximately to 50%–65% HRR) as determined from the 6MWT and verified using heart rate monitoring and Borg RPE.

### 2.7 Blood Analysis

Fasting blood samples were collected in the morning (around 8 a.m.) after an overnight fast of approximately 10–12 h. Samples were taken at three time points: 24 h before the start of the training program, and 48 h after the completion of the training period.

For biochemical analyses, 5 mL of blood was drawn into tubes containing the anticoagulant lithium heparin.

Samples were centrifuged at 2000 × g for 25 min to separate the serum, which was then used to determine concentrations of alanine aminotransferase (ALT), aspartate aminotransferase (AST), alkaline phosphatase (ALP), gamma‐glutamyl transferase (GGT), bilirubin, and C‐reactive protein (CRP). Analyses were performed using a Chemistry System Analyzer (Beckman Coulter AU480, Villepinte, France).

For erythrocyte sedimentation rate (ESR) measurements, blood samples were collected in vacutainer tubes containing sodium citrate and assessed using the Westergren method.

### 2.8 Statistical Methods

All statistical analyses were executed using SPSS statistical package version 27.0 (IBM SPSS Statistics, Chicago, IL, USA). The normal distribution of all variables was first confirmed using the Kolmogorov–Smirnov test. Independent *t*‐tests and Mann‐Whitney U‐tests were performed to test for statistically significant differences between groups for variables at baseline.

A two‐way repeated measures analysis of variance (ANOVA) (2 groups × 2 time points: pre‐ and post‐intervention) with Bonferroni adjustments for multiple comparisons was performed using the absolute values of the outcome variables at both time points. This approach allowed us to assess differences within and between groups over time. Baseline differences between groups were non‐significant; therefore, a two‐way repeated measures ANOVA was applied to assess within‐ and between‐group effects over time. ANCOVA using baseline values as covariates was considered but deemed unnecessary due to non‐significant baseline differences. In addition, comparisons between groups at each time point were conducted using independent *t*‐tests. The effect size of each variable was also reported (Cohen 1988). A two‐tailed significance level was considered, and a *p*‐value less than 0.05 was regarded as statistically significant. No interim analyses were planned, and no formal stopping guidelines were established.

## 3 Results

Mean ± SD values for body composition, liver biomarker, and selected inflammatory markers before‐ and after the MIWT are presented in Table 2. No significant differences between groups (TG and CG) were found at baseline for any measured variable. No significant differences were detected in the CG between baseline and post‐intervention measurements.

**TABLE 2:** Anthropometric and biochemical parameters at baseline and post‐intervention and (time*group) interaction in the training and control groups.

	Control group (*n* = 18)		Training group (*n* = 18)		Interaction (time*group)^a^		
	Pre	Post	Pre	Post	F	*p*‐value	*η*_p_^2^
Age (years)	56.5 ± 3.65		54.8 ± 3.18				
Height (m)	1.59 ± 0.06		1.60 ± 0.07				
Body mass (Kg)	89.6 ± 14.1	89.7 ± 13.7	84.2 ± 9.88	82.3 ± 9.77***	15.7	< 0.001	0.32
Body mass index (Kg/m^2^)	35.1 ± 5.21	35.2 ± 5.14	32.9 ± 4.76	32.2 ± 4.47***	16.1	< 0.001	0.32
Body fat (%)	44.6 ± 5.76	44.6 ± 5.74	44.0 ± 4.57	43.0 ± 4.52**	12.7	0.001	0.27
Waist circumference (cm)	113 ± 10.12	114 ± 10.2	110 ± 10.0	104 ± 8.13***^b^	25.6	< 0.001	0.43
Hip circumference (cm)	115 ± 10.5	116 ± 10.3	111 ± 10.4	108 ± 8.63**^c^	9.1	0.005	0.21
Waist to hip ratio	0.99 ± 0.07	0.99 ± 0.06	0.99 ± 0.06	0.96 ± 0.07*	4.77	0.036	0.12
6 min walking test distance (m)	473 ± 24.6	471 ± 24.4	481 ± 26.1	494 ± 25.7*^b^	5.84	0.021	0.15
ALT (U/L)	24.8 ± 5.94	25.2 ± 5.40	25.3 ± 5.30	23.8 ± 5.17**	4.53	0.041	0.12
AST (U/L)	27.6 ± 6.60	28.3 ± 6.81	28.1 ± 6.15	26.3 ± 6.43*	5.39	0.026	0.14
GGT (U/L)	23.6 ± 7.11	24.6 ± 7.49	22.4 ± 7.40	20.6 ± 7.20*	4.43	0.043	0.12
ALP (U/L)	179.8 ± 21.9	182.6 ± 23.2	184.6 ± 23.9	175.3 ± 27.0	3.85	0.058	0.10
Total bilirubin (umol/L)	9.32 ± 4.21	8.97 ± 3.73	10.6 ± 4.08	11.1 ± 4.54**	4.21	0.048	0.11
Direct bilirubin (umol/L)	2.61 ± 0.65	2.45 ± 0.65	2.55 ± 0.60	2.56 ± 0.53	0.73	0.398	0.02
Indirect bilirubin (umol/L)	6.71 ± 4.24	6.51 ± 3.87	8.02 ± 3.87	8.57 ± 4.47	2.68	0.111	0.07
C‐reactive protein (mg/L)	4.15 ± 1.35	4.24 ± 1.08	4.49 ± 0.88	4.04 ± 1.01**	4.45	0.042	0.12
ESR (mm/h)	18.1 ± 5.38	18.5 ± 5.34	17.7 ± 3.76	16.8 ± 4.22	1.29	0.264	0.04

*Note:* Results are given as means ± SD.

Abbreviations: ALP: alkaline phosphatase; ALT: alanine aminotransferase; AST: aspartate aminotransferase; GGT: gamma‐glutamyltranspeptidase.

^a^ *comparison carried out using two‐way repeated measures ANOVA.*

^b^ *p* *<* *0.001 (compared to post‐intervention value in the control group)*.

^c^ *p* *<* *0.01.*

*p < *0.05*; **p < *0.01*; ***p < *0.001* *(compared to pre‐intervention value in the group)*.

All participants reported no substantial changes in their dietary habits during the study period, indicating that the observed changes in body composition and biochemical parameters were likely attributable to the exercise intervention.

### 3.1 Anthropometric Parameters and Aerobic Capacity

There was a significant (time × groups) interaction on all body composition parameters and 6MWT_distance_. Bonferroni‐adjusted post‐hoc tests revealed significant before to‐after MIWT decrease in body mass (−2.26%, *p* < 0.001), BMI (−2.13%, *p* < 0.001), BF (−2.27%, *p* = 0.001), WC (−5.46%, *P* < 0.001), HC (−2.70%, *p* = 0.005), WHR (−3.03%, *p* = 0.021) and a significant increase of 6MWT_distanc_ (+2.70%, *p* = 0.031). In the between groups difference, the TG noted a significant decrease in WC and HC (*p* < 0.001 and *p* = 0.008, respectively) and a significant increase in 6MWT_distance_ (*p* = 0.010) when compared to the CG.

### 3.2 Biochemical Parameters

A significant (time × groups) interaction effect (time × group) was obtained for ALT (F_(1‐34)_ = 4.53, *p* = 0.041, *η*_p_^2^ = 0.12), AST (F_(1‐34)_ = 5.39, *P* = 0.026, *η*_p_^2^ = 0.14), GGT (F_(1‐34)_ = 4.43, *p* = 0.043, *η*_p_^2^ = 0.12), total bilirubin (F_(1‐34)_ = 4.21, *p* = 0.048, *η*_p_^2^ = 0.11) and CRP (F_(1‐34)_ = 4.45, *p* = 0.042, *η*_p_^2^ = 0.12), indicating that changes over time differed significantly between the training group (TG) and control group (CG).). However, no significant interaction effects were found for ALP (F_(1‐34)_ = 3.85, *p* = 0.058, *η*_p_^2^ = 0.10), direct bilirubin (F_(1‐34)_ = 0.73, *p* = 0.398, *η*_p_^2^ = 0.02), indirect bilirubin (F_(1‐34)_ = 2.68, *p* = 0.111, *η*_p_^2^ = 0.07), or ESR (F_(1‐34)_ = 1.29, *p* = 0.264, *η*_p_^2^ = 0.04).

Post hoc analyses within the TG revealed significant decreases from pre‐to post‐intervention in ALT (25.33 ± 5.30 vs. 23.83 ± 5.17 (U/L), *p* = 0.002, *d* = 0.29[*small*]), AST (28.11 ± 6.15 vs. 26.33 ± 6.43 (U/L), *p* = 0.013, *d* = 0.29[*small*]), GGT (22.39 ± 7.40 vs. 20.61 ± 7.20 (U/L), *p* = 0.036, *d* = 0.25[*small*]) and CRP (4.49 ± 0.88 vs. 4.04 ± 1.01 (mg/L), *P* = 0.007, *d* = 0.49[*small*]), alongside a significant increase in total bilirubin (10.57 ± 4.08 vs. 11.13 ± 4.54 (umol/L), *p* = 0.009, *d* = 0.13[*small*]) No significant differences were detected for any variables in the CG. These results highlight the beneficial effects of the intervention on liver function and inflammation markers in the TG compared to the CG.

## 4 Discussion

To the best of our knowledge, this is the first study investigating changes in liver function and selected inflammatory markers following a 10‐week moderate‐intensity intermittent walking training (MIWT) program in postmenopausal women with obesity.

Our results support the initial hypothesis, demonstrating that MIWT intervention produces statistically significant improvements in specific liver enzymes, inflammatory parameters and body composition in postmenopausal women with obesity. However, it is important to note that while these changes reached statistical significance, effect sizes were generally small, and baseline values were largely within clinical reference ranges, which may limit the immediate clinical impact of these findings.

Specifically, we observed significant decreases in body mass, BMI, body fat percentage, waist circumference, hip circumference, and waist‐to‐hip ratio following the intervention.

In postmenopause, rapid physiological and hormonal changes, such as decreasing estrogen levels, lead to reductions in muscle mass and accelerated fat accumulation (El Khoudary et al. 2020; Davis et al. 2023). Consequently, obesity (Gold et al. 2017) and increased body fat can occur, both of which are recognized risk factors for cardiovascular disease (De Lucia Rolfe et al. 2015).

A growing body of evidence indicates that regular physical activity contributes to reductions in visceral fat and improvements in muscle mass—factors crucial for mitigating the risks associated with metabolic syndrome and cardiovascular disease in this population (Chomiuk et al. 2024; Shariful Islam et al. 2023; Weiss et al. 2017).

Even modest reductions in waist circumference and fat mass, as observed in our study, may have meaningful long‐term implications for metabolic and cardiovascular health, as small improvements in body composition can lead to favorable changes in insulin sensitivity, lipid profile, and systemic inflammation, especially if maintained over time (Son et al. 2023; Chomiuk et al. 2024; Shariful Islam et al. 2023).

Given the well‐established link between increased visceral fat and metabolic dysfunction during menopause, the reduction in waist circumference observed in the intervention group likely reflects physiologically relevant changes. These changes may suggest that exercise can help counteract some of the detrimental metabolic effects associated with the menopausal transition (Kodoth et al. 2022; Mika et al. 2019). These findings highlight the role of MIWT in the management of menopause‐related weight gain and are consistent with previous research demonstrating that walking programs improve body composition in sedentary postmenopausal women with obesity (Son et al. 2023; Guzel et al. 2024).

Although ALT and AST are key hepatic enzymes influenced by obesity and menopause (Chung et al. 2015; McGill 2016), they are not specific indicators of these conditions. ALT is mainly found in hepatocyte cytoplasm, while AST is present in both cytoplasm and mitochondria; elevations indicate hepatocyte membrane or organelle damage (McGill 2016). The reductions in ALT and AST observed in this study were small and remained within normal clinical ranges, indicating modest changes in liver‐related biomarkers rather than direct improvements in clinical liver function.

Structured exercise, including aerobic and resistance training, has been shown to reduce ALT and AST levels (Hejazi and Hackett 2023; Sabag et al. 2022; Wang et al. 2020; El‐Eshmawy 2023; Xiong et al. 2021; Zhou et al. 2021; Keating et al. 2012; Smart et al. 2018; Shi et al. 2025; Farhadi et al. 2020; Yoshimura et al. 2014), though results vary across populations and exercise modalities (Park et al. 2019; Moro et al. 2020; Cassidy et al. 2016).

For example, aerobic training is often effective in reducing ALT, while resistance training can additionally reduce AST (El‐Eshmawy 2023). Meta‐analyses suggest aerobic exercise may be more effective than resistance or high intensity training (Xiong et al. 2021), whereas combined exercise modalities may produce the most favorable changes in ALT, AST, and other metabolic markers (Zhou et al. 2021). However, some studies report no significant improvements in these enzymes (Keating et al. 2012; Smart et al. 2018). Differences in outcomes are likely related to participant characteristics, body composition, and exercise volume, with higher‐volume moderate‐intensity aerobic training generally more effective (Katsagoni et al. 2017).

This is the first study to evaluate the effect of a 10‐week moderate‐intensity aerobic exercise training (MIWT) program on liver markers in postmenopausal women with obesity. The significant improvements observed in ALT and AST are consistent with previous studies reporting that 24 and 12 weeks of aerobic exercise training significantly reduced levels of these markers in obese menopausal (Farhadi et al. 2020) and middle‐aged women (Yoshimura et al. 2014). Aerobic training and resistance training were all effective in reducing ALT, while only resistance exercise training also led to reductions in AST levels. However, some studies report no significant changes in ALT or AST after shorter or alternative exercise protocols (Park et al. 2019; Moro et al. 2020; Cassidy et al. 2016), likely due to differences in participant characteristics (age, BMI, sex), body composition, exercise modality, and training volume. The effects of exercise on aminotransferases are often linked to weight loss, which reduces inflammatory activity and hepatic fat (Brea and Puzo 2013). Higher‐volume moderate‐intensity aerobic training (> 180 min/week) generally produces greater improvements in liver enzymes than lower‐volume programs (Katsagoni et al. 2017). Potential mechanisms include enhanced insulin sensitivity, increased hepatic fat oxidation, reduced lipogenic enzyme activity, and decreased intrahepatic fat accumulation (Warner et al. 2020; Samuel and Shulman 2018; Matsuzaka and Shimano 2011). Although these mechanisms were not directly measured in this study, they are supported by prior evidence. Future research should include biochemical and imaging assessments to further clarify these physiological processes.

GGT, a marker associated with hepatic lipid accumulation and oxidative stress (Kawamoto et al. 2009), was also reduced following MIWT. Although effect sizes were small, this reduction may reflect early favorable adaptations that could contribute to long‐term hepatic health (Pajuelo‐Vasquez et al. 2024). Although limited research has been conducted on the effects of walking training on serum GGT, several studies on aerobic, resistance, and combined training modalities have reported beneficial effects on GGT levels in overweight, obese, and postmenopausal women (Barsalani et al. 2013; Skrypnik et al. 2016; Reverter‐Masia et al. 2021).

Serum ALP, a marker of hepatobiliary function and chronic low‐grade inflammation (J. H. Kim et al. 2020), did not significantly change following the 10‐week MIWT program. This aligns with findings by Rezende et al. (Rezende et al. 2016), who reported no change in ALP after 24 weeks of aerobic training in postmenopausal women. In contrast, another study observed increased ALP after 12 weeks of aerobic exercise in overweight women (Tartibian et al. 2018). These discrepancies may be due to differences in training protocols and participant characteristics. Given that ALP is influenced by multiple tissues beyond the liver, its response to moderate exercise may be less pronounced or require longer durations to manifest.

While ALP may be less responsive to moderate exercise, C‐reactive protein (CRP) is a more sensitive marker of systemic inflammation and appears to respond readily to exercise‐induced changes (Gleeson et al. 2011). In the present study, a 10‐week MIWT program significantly reduced CRP levels in obese postmenopausal women, despite baseline values being within normal ranges. Even modest reductions in CRP are clinically meaningful because low‐grade systemic inflammation, as reflected by CRP, contributes to atherogenesis, insulin resistance, and cardiometabolic risk; reductions in CRP have been associated with lower incidence of cardiovascular events and improved metabolic profiles in populations at risk, independent of baseline CRP levels (Ridker et al. 2000).

These findings align with systematic reviews and meta‐analyses indicating that aerobic, resistance, and combined exercise programs longer than 8 weeks effectively lower CRP in postmenopausal women with overweight or obesity (Tan et al. 2023; Abd El‐Kader and Al‐Jiffri 2019). However, shorter or lower‐intensity interventions, such as 8 weeks of moderate walking, may not produce significant changes (Özdemir et al. 2021).

Our results support the growing body of evidence suggesting that aerobic exercise, including moderate walking, may play a role in alleviating low‐grade chronic inflammation in postmenopausal women with obesity (Hayashino et al. 2014). The exact biological mechanisms are not yet fully elucidated but may include reduced body fat mass and macrophage infiltration in adipose tissue, increased production of interleukin‐6 (IL‐6) as an anti‐inflammatory cytokine, suppression of tumor necrosis factor‐alpha (TNF‐α), and activation of the cholinergic anti‐inflammatory pathway (Woods et al. 2006). Additionally, exercise‐induced changes in nitric oxide and reactive oxygen species may contribute to the modulation of intracellular signaling pathways involved in inflammation (Scheele et al. 2009).

Bilirubin, the end product of heme catabolism, is a potent antioxidant that may reduce hepatic fat accumulation and inflammation (Shu et al. 2022). In the present study, a 10‐week MIWT program significantly increased total bilirubin levels, with no changes in direct or indirect bilirubin. This is consistent with previous findings showing elevated total bilirubin following prolonged aerobic training in sedentary postmenopausal women (Swift et al. 2012), whereas other training modalities in middle‐aged women with obesity did not affect bilirubin (Skrypnik et al. 2016). The increase in total bilirubin observed in this study may be associated with exercise‐induced hemolysis, as suggested in previous research, potentially triggered by elevated core temperature and oxidative stress (Flack et al. 2023), which has been proposed to enhance heme catabolism and antioxidant capacity (S. Y. Kim and Park 2012). Elevated bilirubin could therefore contribute to protection against hepatic fat accumulation and inflammation, although these mechanisms were not directly assessed in the present study (Kunutsor et al. 2020).

In the present study, erythrocyte sedimentation rate (ESR), a non‐specific marker of systemic inflammation, did not change after 10 weeks of MIWT. This aligns with previous evidence suggesting that aerobic training alone may not significantly affect ESR (Hu et al. 2021). However, higher‐intensity interventions, such as a 10‐week high‐intensity interval walking program, have been shown to reduce ESR in older adults (Bartlett et al. 2018), indicating that exercise intensity may influence this marker.

Overall, while the statistically significant changes observed in liver enzymes, inflammatory markers, and body composition were modest and within reference ranges, they demonstrate that MIWT is a feasible, safe, and physiologically beneficial intervention for postmenopausal women with obesity. These early adaptations provide a foundation for future studies that could combine MIWT with nutritional guidance or longer‐term interventions to further enhance clinical relevance.

Due to its safety and feasibility, MIWT can be easily implemented in community or outpatient settings, promoting adherence and long‐term benefits. Future research could explore combining MIWT with nutritional guidance and metabolic monitoring to further optimize outcomes in postmenopausal women with obesity.

The present study had some limitations that deserve attention. First, the findings are limited by the absence of imaging‐based liver fat assessment, insulin resistance markers, and broader inflammatory profiling, as only standard CRP and ESR were measured; other relevant cytokines such as TNF‐α, interleukin‐2, interleukin‐4, and interleukin‐6 were not assessed. Another limitation of this study is the use of standard CRP instead of high‐sensitivity CRP (hsCRP) to assess systemic inflammation. Nutritional intake was not strictly controlled, although participants were instructed to maintain their usual diet, and no substantial changes were reported. Nutritional intake was not strictly controlled, although participants were instructed to maintain their usual diet and no substantial changes were reported. While formal dietary control could have strengthened the study, the primary aim was to evaluate the effects of moderate‐intensity intermittent walking training (MIWT) on liver enzymes, inflammatory markers, and body composition under real‐life, free‐living conditions. This approach enhances the external validity and practical applicability of the findings, providing insight into a feasible intervention for postmenopausal women with obesity.

Finally, the restricted sample of healthy obese postmenopausal women without diagnosed liver disease limits the generalisability of the findings. A larger and more diverse sample, potentially including individuals with NAFLD or other metabolic conditions, is needed to validate and generalize these results.

Further research should also investigate the impact of moderate walking on quality of life additional biochemical and functional outcomes, and the underlying mechanisms linking exercise to hepatic and metabolic adaptations.

## 5 Conclusions

The MIWT intervention produced statistically significant improvements in body composition and liver‐related biomarkers, including ALT, AST, GGT, bilirubin, and CRP, in postmenopausal women with obesity. These findings indicate that MIWT is associated with favorable changes in biochemical markers related to metabolic and inflammatory status in this population. The observed changes should be interpreted as short‐term physiological adaptations in liver‐related and inflammatory biomarkers following a moderate‐intensity walking intervention. Accordingly, MIWT represents a feasible and well‐tolerated lifestyle approach capable of inducing modest but measurable changes in cardiometabolic‐related markers, body composition, and aerobic capacity.

These results add to the growing body of evidence supporting the role of structured physical activity in modulating metabolic and inflammatory risk profiles in postmenopausal women with obesity. Future studies should investigate the long‐term sustainability of these adaptations, include direct assessments of liver structure and function (e.g., imaging or functional tests), and evaluate clinically meaningful outcomes to define the translational relevance of these early biomarker changes.

## Funding

The authors have nothing to report.

## Conflicts of Interest

The authors declare no conflicts of interest.

## Data Availability

The data that support the findings of this study are available on request from the corresponding author. The data are not publicly available due to privacy or ethical restrictions.
